# Impact of postoperative patient-prosthesis mismatch as a risk factor for early structural valve deterioration after aortic valve replacement with Trifecta bioprosthesis

**DOI:** 10.1186/s13019-022-01918-3

**Published:** 2022-07-08

**Authors:** Tatsuto Wakami, Shigeki Koizumi, Tadaaki Koyama

**Affiliations:** Department of Cardiovascular Surgery, Kobe City Medical Center General Hospital, 2-1-1 Minatojima Minamimachi Chuoku Kobeshi Hyogoken, Kobe, 650-047 Japan

**Keywords:** Trifecta, Aortic valve, Structural valve deterioration

## Abstract

**Background:**

Several studies have reported high rates of structural valve deterioration (SVD) in the Trifecta valves. Herein, we analyzed the midterm results of the Trifecta valve and risk factors for early SVD.

**Methods:**

We retrospectively reviewed the records of 110 patients who had undergone Trifecta implantation between January 2012 and December 2017.

**Results:**

We encountered seven cases of Trifecta valve failure. We performed a redo aortic valve replacement in five patients and a transcatheter aortic valve replacement in two patients. The SVD rate was 4.8% at 5 years and 6.6% at 7 years. The mean pressure gradient and peak velocity on the first postoperative echocardiogram in patients with SVD were higher than those in patients without SVD. The SVD rates with and without patient-prosthesis mismatch (PPM) were 2.8% and 12.6% at 5 years and 2.8% and 20.0% at 7 years. PPM is a risk factor for SVD. Noncoronary cusp tears were observed in all patients who had undergone redo surgery.

**Conclusions:**

The most common cause of SVD was noncoronary cusp tear. Patients with PPM are at high risk of developing SVD.

## Background

The Trifecta valve (Abbott Vascular, Santa Clara, CA, USA) was introduced for commercial use in 2010 and approved in Japan in 2012. Excellent hemodynamic performance and durability have been reported during midterm follow-up [1, 2]. The excellent hemodynamics are due to the expansive valve design with a bovine pericardial sheet externally mounted on a titanium stent. However, recent studies have reported early structural valve deterioration (SVD) in leaflet tears [2–12]. Fukuhara et al. reported that the rate of SVD was higher in the Trifecta group (n = 508) than in the non-Trifecta group (n = 550) (13.3% vs 4.6%; P = 0.010) [13]. However, the cause and risk factors for early SVD are unknown.

Japanese patients have smaller body sizes and aortic annuli than those of Western patients [14]. Therefore, we used a small Trifecta at a high rate to avoid a patient-prosthesis mismatch (PPM). There are limited reports of valve durability and SVD of small Trifecta implanted in small aortic annuli in Japanese patients. In this study, we analyzed the midterm results of the Trifecta valve and risk factors for early SVD.

## Methods

### Patients’ data

We retrospectively reviewed the data of 110 consecutive patients who had undergone implantation of Trifecta at the Kobe City Medical Center General Hospital between January 2012 and December 2017. The institutional review board approved this study (No. zn210902). Informed consent was obtained from all patients. All patients underwent transthoracic echocardiography preoperatively and one week after Trifecta valve implantation at predischarge. Follow-up echocardiography was performed annually, and all patients, except for one patient who suffered from hospital death, underwent at least one echocardiographic assessment in outpatient. Final follow-up data were collected via the medical records system survey in our institution. Mean follow-up time, defined as the interval between the surgery and the last outpatient visiting or death, was 66 months. The follow-up rate was calculated the proportion of the patients at baseline who remained through the end of the study interval or developed the event of interest by the end of the interval [15]. All-cause mortality and SVD incidence were evaluated in 110 of these patients. Among the patients, 7 patients were suffered from SVD. We divided the SVD group (n = 7) and the non-SVD group (n = 103) and assessed the risk factor of SVD.

### Surgical techniques

Three surgeons, not including trainees performed the surgeries. The choice of the prosthetic valves was left to the discretion of the surgeons. The valves were implanted using standard methods of a full median sternotomy approach in all patients. The Trifecta valves were implanted in a supra-annular position using interrupted horizontal mattress pledgeted suture or intra-annular position using simple interrupted sutures, depending on the surgeon's preference. In the case of tricuspid or type1 or 2 bicuspid, the bioprosthesis was implanted so that the commissures of the native and the prosthesis coincided. In the case of type0, we implanted valve so that the coronary ostium and stent post did not interfere with each other. We tied the sutures with fingers and kept a holder on the prosthesis. All patients were administered antiplatelet therapy and anticoagulant therapy for three months after implantation the Trifecta valve.

### Definition

SVD was defined as a mean transvalvular gradient > 40 mmHg, an increase in the mean transvalvular gradient > 20 mmHg, severe intra-prosthetic aortic regurgitation, or a new or worsening (> 2+/4+) gradient (> 2+/4+) from baseline [16]. Prosthetic valve endocarditis, valve thrombosis, PPM without loss of valve function, and isolated paravalvular leak were not considered SVD. PPM was defined as effective valve orifice index (EOAI) < 0.85 cm^2^/m^2^.

### Statistical analysis

Continuous variables were presented as means ± SDs, and categorical variables were presented as proportions and absolute numbers. The differences between the groups were analyzed using an unpaired t-test or the Mann–Whitney U test for continuous variables and a χ^2^ test or Fisher exact test for categorical variables. Risk factors for the longitudinal data were analyzed using multivariable Cox proportion hazard model. Survival and SVD rates were evaluated using the Gray k-sample test. P-values less than 0.05 were considered statistically significant. Time to event was determined as the number of months between the date of operation and that of follow-up or the date of death. Statistical analyzes were performed using the JMP software (version 14.1.0; SAS Institute, Cary, NC, USA).

## Results

### Patient characteristics and operative data

The clinical characteristics of the patient and the surgical data are shown in Tables 1 and 2, respectively. Valve lesions in the first valve replacement included aortic stenosis (AS) (n = 77), aortic regurgitation (AR) (n = 21), and aortic stenosis and regurgitation (ASR) (n = 12). Concomitant surgeries included mitral valve or tricuspid valve surgery (n = 32), coronary artery bypass grafting (n = 22), and others (n = 36). The sizes of the prostheses label were 19 (n = 47), 21 (n = 44), 23 (n = 12), and 25 mm (n = 7). The Trifecta valves were implanted in the supra-annular (n = 30) and intra-annular (n = 70) positions.

**Table 1:** Clinical characteristics of patients

Valuable, mean ± SD, n (%)	Early SVD (n = 7)	No SVD (n = 103)	P value
Age, years	74.9 ± 7.8	78.3 ± 5.0	0.26
Age > 70	5 (71)	98 (95)	0.33
Male	2 (29)	49 (48)	0.33
BSA	1.51 ± 0.20	1.54 ± 0.18	0.61
Hypertension	6 (86)	73 (71)	0.4
Diabetes	1 (14)	28 (27)	0.45
Dyslipidemia	4 (57)	37 (36)	0.26
Ischemic heart disease	1 (14)	7 (7)	0.46
Dialysis	0 (0)	1 (1)	0.93
Left ventricular ejection fraction < 50%	1 (14)	10 (10)	0.53
Bicuspid aortic valve	1 (14)	8 (8)	0.8
Annulus size	20.1 ± 1.5	20.9 ± 2.5	0.47
Valsalva size	30.1 ± 2.0	32.5 ± 4.1	0.14
STJ size	25.3 ± 3.3	26.0 ± 4.0	0.63
CPB time, min	196 ± 80	168 ± 62	0.25
Cross-clamp time, min	139 ± 75	114 ± 46	0.18
Valve size			0.4
19 mm	5 (71)	42 (41)	0.13
21 mm	2 (29)	42 (41)	
23 mm	0 (0)	12 (12)	
25 mm	0 (0)	7 (7)	
Supra-annular position	3 (43)	27 (26)	0.35
Concomitant procedures			
Coronary artery bypass grafting	1 (14)	21 (20)	0.69
Mitral/tricuspid	3 (43)	29 (28)	0.41
Others	2 (29)	34 (33)	0.93

Data are expressed as mean ± SD or n (%)

SD, Standard deviation; SVD, structural valve deterioration; BSA, body surface area; STJ, sino-tubular junction; CPB, cardiopulmonary bypass

**Table 2:** Operative data and first echocardiographic data after Trifecta valve implantation

Valuable, mean ± SD, n (%)	Early SVD (n = 7)	No SVD (n = 103)	P value
ICU stay, day	3.1 ± 1.2	4.4 ± 4.0	0.2
30 days mortality	0 (0)	1 (1)	0.79
Postoperative TTE			
LVEF	63.5 ± 11.5	57.3 ± 10.0	0.11
Peak velocity, m/s	2.54 ± 0.36	2.11 ± 0.44	0.01
Mean pressure gradient, mmHg	13.9 ± 3.8	9.5 ± 4.1	0.01
EOAI, cm^2^/m^2^	0.77 ± 0.19	0.96 ± 0.22	0.03
PPM (EOAI < 0.85)	5 (71)	20 (22)	0.01

Data are expressed as mean ± SD or n (%)

ICU, intensive care unit; TTE, transthoracic echo; LVEF, left ventricular ejection fraction; EOAI, effective orifice area index; PPM, patient-prosthesis mismatch

### Follow-up results

**Fig. 1:**
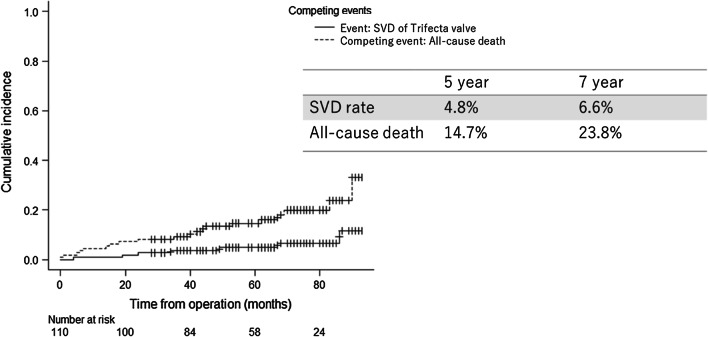
Cumulative incidence of Trifecta SVD. The competing event was death by all causes. SVD, structural valve deterioration

The mean follow-up duration was 66 months. The clinical follow-up rate was 92.7%. Figure 1 shows the all-cause death and SVD rates. The five-and seven-year all-cause death rates were 14.7% and 23.8%, respectively. Seven patients (6.4%) had SVD and all required repeat surgeries. The SVD rate was 4.8% at 5 years and 6.6% at 7 years.

### Clinical characteristics of the SVD group

Table 3 presents the clinical details of the SVD group. Mean valve durability was 40 ± 27 months.

**Table 3:** Clinical details of patients with Trifecta valve structural valve deterioration

No.	Age (years), sex	Valve size (mm)	Postoperative EOAI (cm^2^/m^2^)	Durability (month)	mPG at last follow-up TTE (mmHg)	AR at last follow-up TTE	Redo Indication	Redo Surgery	Details
1	72, F	19	0.82	4	14	3	AR	SAVR	Cusp tear and pannus
2	76, F	19	0.78	19	29	4	AR	SAVR	Cusp tear
3	76, M	21	0.94	24	26	4	AR	SAVR	Cusp tear and pannus
4	79, F	19	0.64	34	18	4	AR	SAVR	Cusp tear
5	77, M	21	0.52	49	15	3	AR	SAVR	Cusp tear and attachment to Valsalva sinus
6	65, F	19	0.57	67	28	3	AsR	TAVR	
7	79, F	19	1.1	86	54	2	AS	TAVR	

F, female; M, male; mPG, mean pressure gradient; AR, aortic regurgitation; TTE, transthoracic echo; AsR, aortic stenosis and regurgitation; AS, aortic stenosis; SAVR, surgical aortic valve replacement; TAVR, transcatheter aortic valve replacement

The primary pathologies of the aortic valve were AS (n = 6) and ASR (n = 1). The size of the Trifecta valves were 19 mm (n = 5) and 21 mm (n = 2). The indications for SVD reoperation were AS (n = 1), AR (n = 5), and AsR (n = 1). Five patients had redo surgical aortic valve replacements (SAVR) with another prosthesis. No complications occurred. Two patients underwent transcatheter aortic valve replacement without any complications.

### Comparison between SVD and non-SVD

Tables 1 and 2 show comparisons of baseline and operative characteristics between the seven patients with SVD and the other patients. The mean pressure gradient and peak velocity was higher and EOAI was lower in patients with SVD than in patients with no SVD. The rate of patients with postoperative PPM was significantly higher in SVD group than that in non-SVD group. There were no significant differences in the other perioperative variables between the SVD and no-SVD groups. The multivariable risk analysis estimate revealed the rate of PPM was higher in SVD group than non-SVD group (Table 4).

**Table 4:** Multivariable analysis risk estimate of structural valve deterioration

	Multivariable analysis risk estimate, 95% confidence interval	P value
Age > 70	1.21 [0.14–10.6]	0.86
PPM	7.73 [1.46–41.0]	0.02
19 mm valve size	2.42 [0.47–12.6]	0.29

PPM, patient-prosthesis mismatch

### Comparison between PPM and non-PPM

**Fig. 2:**
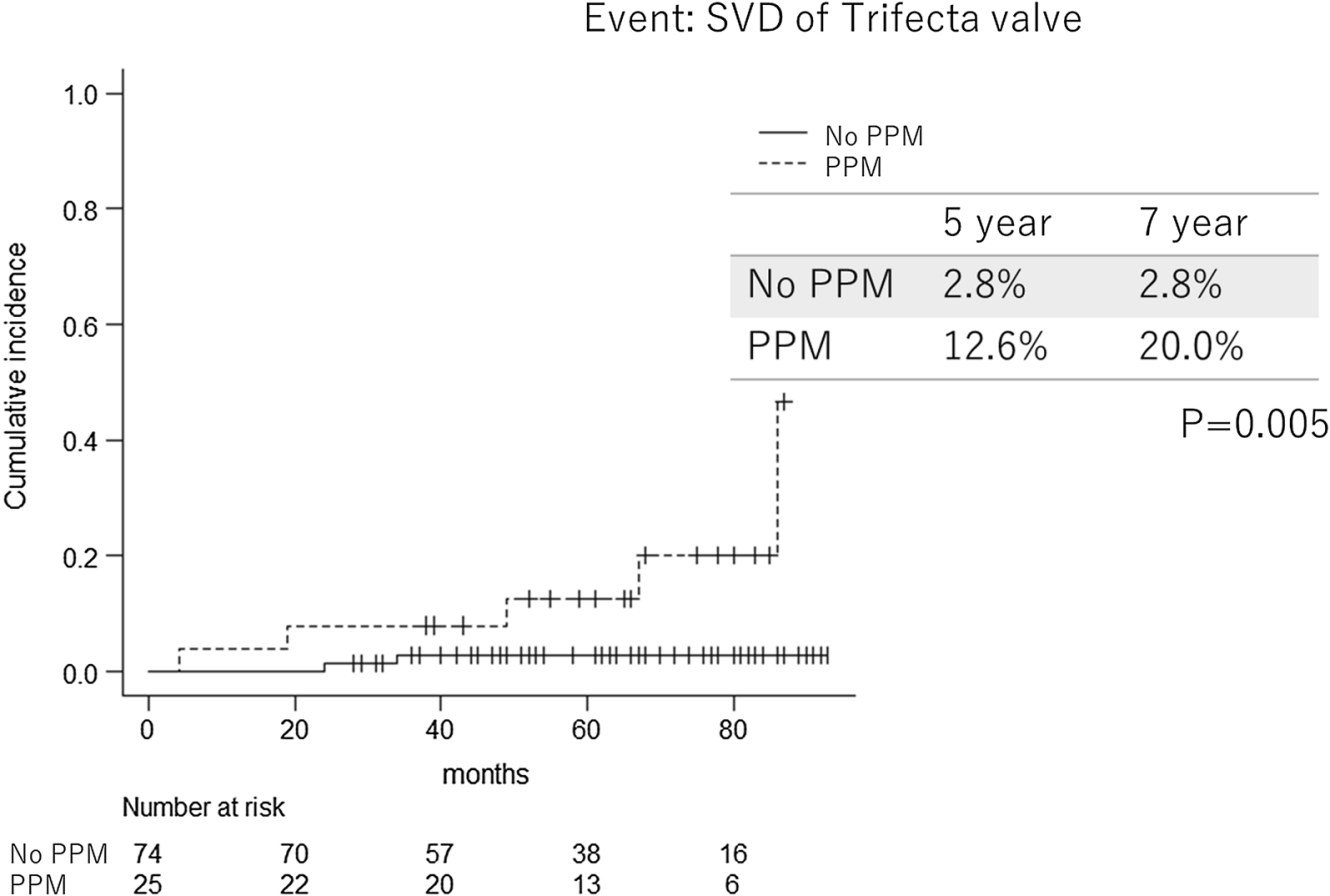
Rate of Trifecta valve failure, comparison between PPM and non-PPM. SVD, structural valve deterioration; PPM, patient-prosthesis mismatch+

Figure 2 shows the SVD rate of the PPM group in postoperative transthoracic echo (n = 25) and non-PPM(n = 74). The SVD rate of non-PPM group and PPM group were 2.8% and 12.6% at 5 years and 2.8% and 20.0% at 7 years. The SVD rate of PPM group was significantly higher than non-PPM group (p = 0.005).

### Comparison between 19 mm versus other valves

**Fig. 3:**
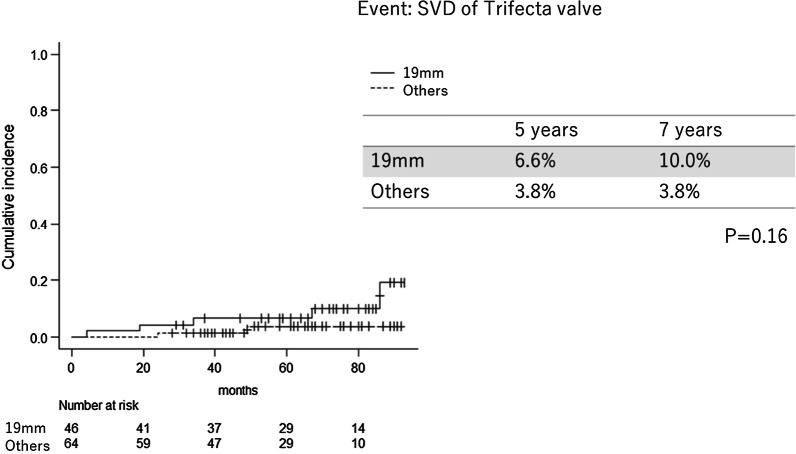
Rate of Trifecta valve failure, comparison between 19 mm valve versus others

Figure 3 shows the SVD rates of the 19 mm valves (n = 47) and other valves (n = 63). The SVD rate of the 19 mm and other valves were 6.6% and 3.8% at 5 years and 10.0% vs. 3.8% at 7 years, respectively. The postoperative mean pressure gradient of the 19 mm valves and other valves was 10.7 ± 3.5 mmHg and 9.2 ± 4.6 mmHg(p = 0.06). The postoperative EOAI of the 19 mm valves are significantly lower than other valves (0.89 ± 0.18 cm^2^/m^2^ vs 0.98 ± 0.25 cm^2^/m^2^, p = 0.04).There were no significant differences in SVD rates between the 19 mm valve group and the other group.

### Gross pathological findings

We evaluated the failure valves of five patients who underwent SAVR pathologically. The cause of AR disorder was a cusp prolapse caused by a tear in a leaflet along with the stent post in all patients. The cusp was detached from the stent post at the commissure of the right coronary cusp (RCC) and the noncoronary cusp (NCC) (n = 4), left coronary cusp (LCC), NCC (n = 1), or RCC and LCC (n = 1). In one patient, the cusp was detached from the RCC-NCC and RCC-LCC commissure.

**Fig. 4:**
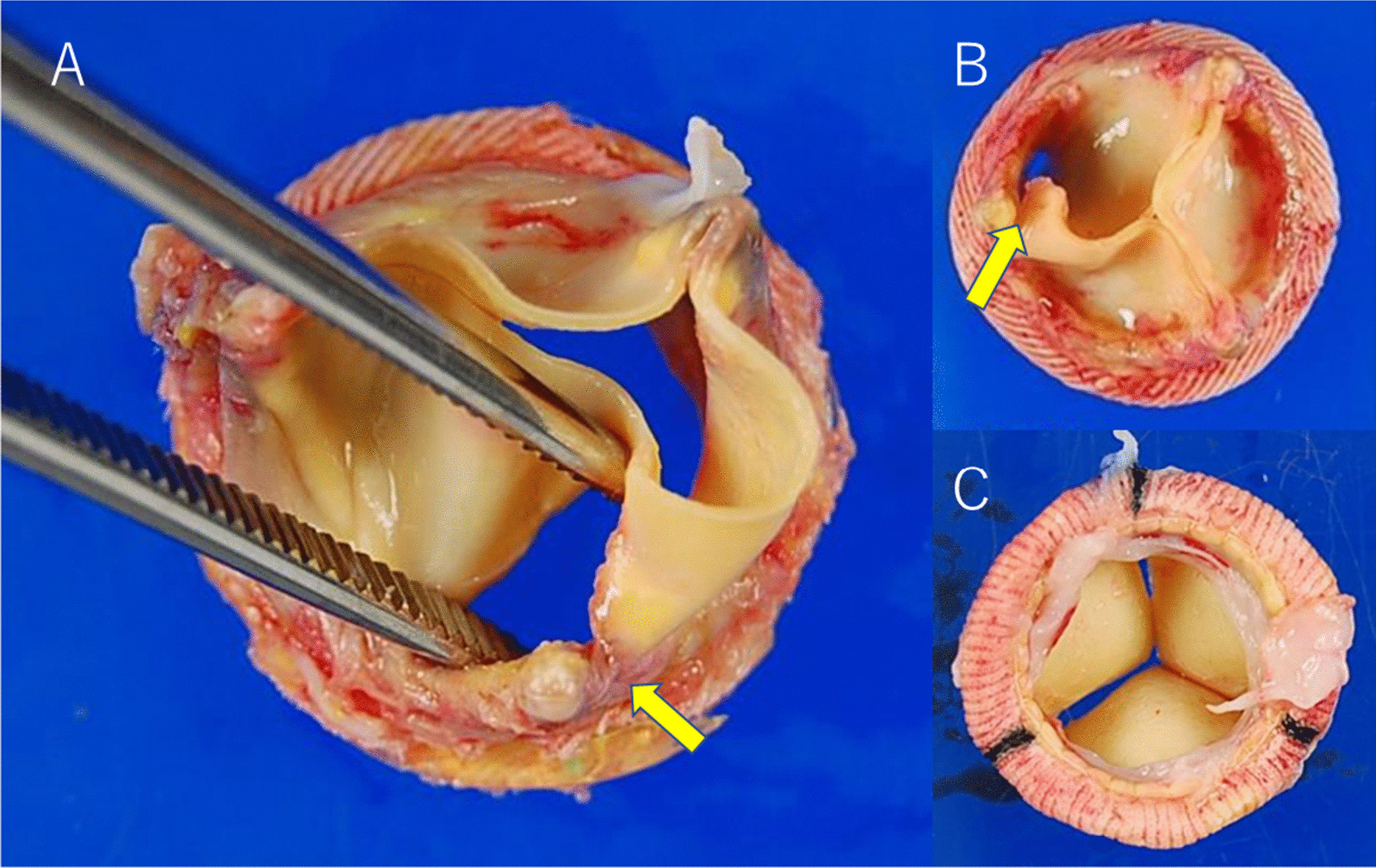
Photographs of Trifecta valves (Abbott Vascular, Santa Clara, CA). **A** 81-year-old man with moderate-severe aortic regurgitation. The durability of the valve was 49 months. There was a large tear at the stent post between the noncoronary cusp and right coronary cusp. **B** 77-year-old woman with severe aortic regurgitation. The durability of the valve was 19 months. There was a partial tear of the noncoronary cusp. **C** 72-year-old woman with severe aortic regurgitation. The durability of the valve was 4 months. The formation of the subvalvular pannus was observed

In one patient, the RCC-LCC and LCC-NCC commissures adhered to the sinus of Valsalva, and there was a tear in the RCC-NCC commissure on its contralateral side, and a Pannus formation was seen under the valve in two patients (Fig. 4).

## Discussion

We reported seven cases of early SVD among 110 Trifecta valve implantations performed between 2012 and 2017 at our center. In our study, the free rate of SVD was 93.4% at seven years, similar to results of other reports [13].

Previous studies showed that a high postoperative mean pressure gradient and PPM are related to SVD [17–19]. In our study, the mean pressure gradient and peak velocity in the first echocardiography were higher in patients with SVD. Patients with a high mean pressure gradient and peak velocity should be closely monitored. The most common cause of SVD was a noncoronary cusp tear. Previous studies have reported similar pathological findings. This study does not show why NCC tear is common. We need more cases to see if this trend is correct and what the causes are. Subvalvular Pannus formations were observed in two patients. Pannus forms due to surgical injury leading to thrombus formation, release of cytokines, and deposition of inflammatory cells [20]. Two patients with pannus formations in our study underwent reoperation for AR. Excessive formation of pannus may confer hemodynamic stress to the leaflet.

In one patient, the RCC-LCC and LCC-NCC commissures adhered to the sinus of Valsalva. Attachment to the Valsalva sinus restricts leaflet movement and incomplete leaflet coaptation. It is probable that the externally mounted leaflet design of the Trifecta valve leads to the attachment of the Valsalva sinus, resulting in limited leaflet motion and valve insufficiency. A previous report showed the same pathological findings [3] that a small aortic root was predisposing factor. Cleveland et al. reported that oversizing of bioprosthetic valves resulted in an increased pressure gradient, and the Trifecta valve was more sensitive to oversizing than other bioprosthetic valves due to the externally mounted leaflet design [21]. Implanting the oversized Trifecta valve in the small annulus may interfere with the expansion of the bioprosthesis, narrowing the EOAI, and creating accelerated blood flow. Moreover, implanting the Trifecta in a small sinus of Valsalva may stress the outer-mounted valve. We used Trifecta valves in older adults, especially in patients with small annuli. Thus, we used 19 mm valves with a higher frequency (43%) than those used in previous studies [13, 22]. In our study, Implanting 19 mm valves showed significantly lower EOAI than other valves. Although implanting 19 mm valves had hemodynamically disadvantage, it did not significantly increase SVD. PPM on postoperative echocardiography was seen in 25 patients (27%). The valves might have been implanted in patients with relatively small body size, in anticipation of favorable hemodynamics of the external annular valve. If the annulus is small and the patient is relatively large and is expected to be PPM, it may be better to consider annulus enlargement rather than using an externally mounted leaflet valve.

This study had several limitations. Our analysis was retrospective and limited to a few patients. Although multivariate analysis is necessary to analyze the risk factors, statistically valid multivariate analysis was difficult due to our small sample size. More well-designed and large-sized studies are essential to understand the mechanism of early Trifecta valve failure. Follow-up echocardiographic studies were performed in various clinical settings.

## Conclusions

In conclusion, although the midterm outcome of the Trifecta valve is acceptable, some patients need redo surgeries due to early SVD. The most common cause of SVD was noncoronary cusp tear. Patients with postoperative PPM were at a high risk of SVD, so they should be closely followed.

## Data Availability

The datasets used and/or analysed during the current study are available from the corresponding author on reasonable request.
